# Supplementary material for: Association between oral microbiome and seven types of cancers in East Asian population: a two-sample Mendelian randomization analysis
Source: Front Mol Biosci. 2023 Nov 21;10:1327893. doi: 10.3389/fmolb.2023.1327893 (PMC10702768; doi:10.3389/fmolb.2023.1327893)
Supplement: Supplementary file 1 [file DataSheet1.ZIP › Supplementary Materials/MR plots for tongue/tongue═╝/Prostate cancer/pheno.253_to_prostate cancer_forest.pdf]

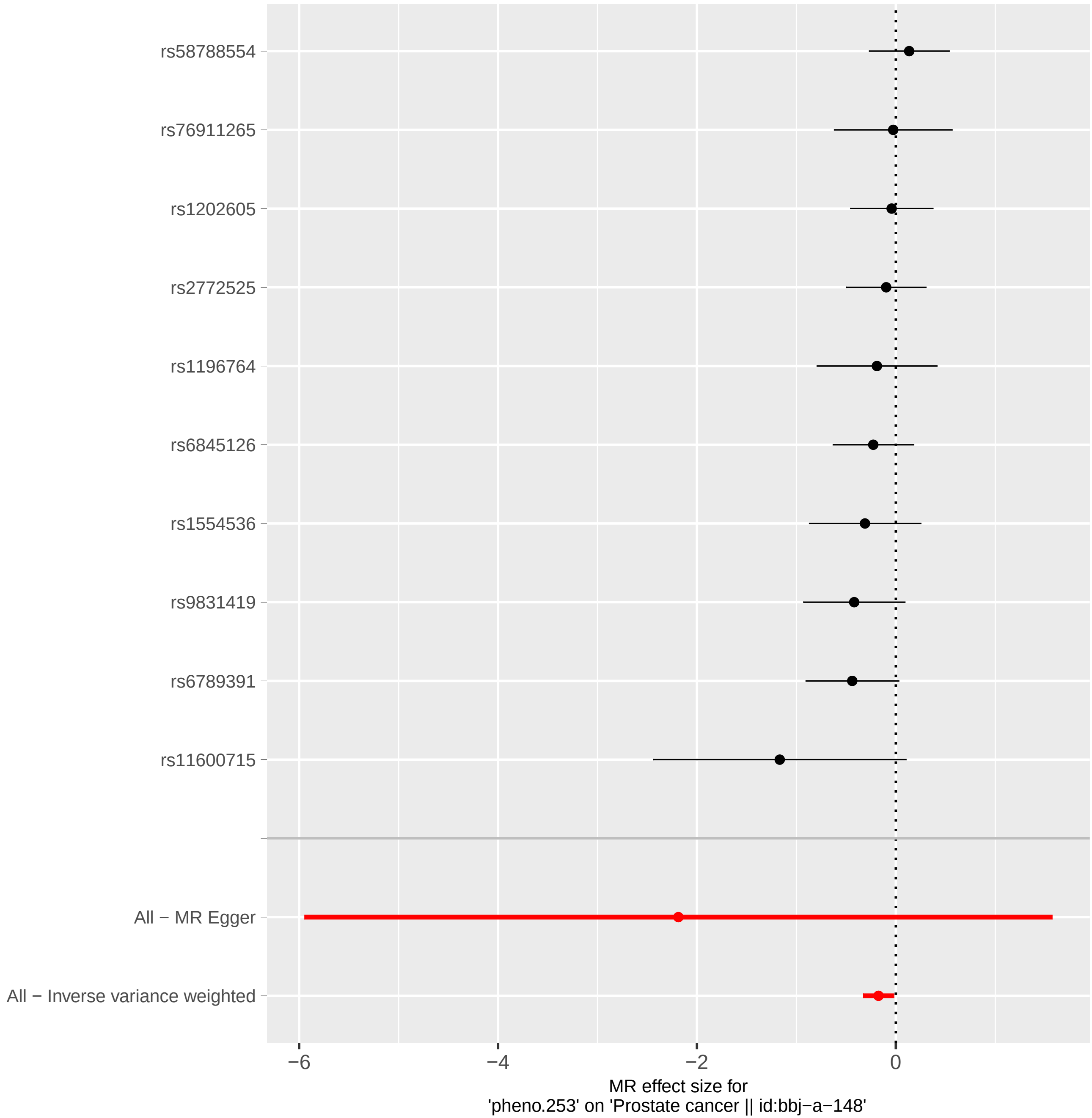
rs58788554
rs76911265
rs1202605
rs2772525
rs1196764
rs6845126
rs1554536
rs9831419
rs6789391
rs11600715
All − MR Egger
All − Inverse variance weighted
−6
−4
−2
0
MR effect size for
'pheno.253' on 'Prostate cancer || id:bbj−a−148'
